# The impact of physical exercise on medical expenditure among middle-aged and older adults: digital literacy as a moderating variable

**DOI:** 10.3389/fpubh.2026.1822634

**Published:** 2026-05-08

**Authors:** Jiaqi Hao, Zongbao Yu, Yifan Zhang, Gang Chen

**Affiliations:** 1 Center for Sports Strategy and Policy Research, Wuhan Sports University, Wuhan, China; 2 Yunnan Normal University School of Physical Education, Kunming, Yunnan, China

**Keywords:** digital literacy, medical expenditures, mental health, physical exercise, physical health

## Abstract

**Introduction:**

As the global population continues to age, the physical and mental health of middle-aged and older adults, along with their medical expenditures, has increasingly drawn societal attention. This study examines the relationship between physical exercise among middle-aged and older adults and their medical expenditures, aiming to provide scientific evidence for improving their quality of life and reducing medical expenditures.

**Methods:**

On the basis of data from the 2020 to 2022 China Family Panel Studies (primarily self-reported or household member-reported data), this research uses baseline regression, mediating effects and moderating effects to explore the impact of physical exercise on medical expenditures among middle-aged and older adults.

**Results:**

The results of this study revealed the following: (1) physical exercise significantly suppresses medical expenditures among middle-aged and older adults, a finding that remains robust after multiple sensitivity checks; (2) physical exercise reduces medical expenditures by promoting physical and mental health in this demographic group; (3) digital literacy enhances the inhibitory effect of physical exercise on medical expenditures, with digital professional literacy being the only dimension to exhibit a significant moderating effect; and (4) the moderating effect of digital literacy is significant among males, urban residents, and those with higher education levels but not among females, rural residents, or those with lower education levels.

**Discussion:**

This study not only contributes to the systematic identification and scientific evaluation of the potential economic benefits of physical exercise within the framework of health economics, thereby providing a scientific foundation for the health management and medical expenditures of middle-aged and older adults but also expands the interdisciplinary research perspective at the intersection of health economics and digital governance, enriching the theoretical explanatory framework in these fields.

## 1 Introduction

In recent years, population aging has become a critical global concern. With its accelerating aging process and massive population, this issue holds even greater significance in China. According to the 2024 National Development Bulletin on Aging Affairs released in August 2025, by the end of 2024, China's population aged 60 and above reached 310.31 million, accounting for 22% of the total population. Among them, those aged 65 and above numbered 220.23 million, representing 15.6% of the total population (1). Concurrently, the older adults population exhibits widespread characteristics such as empty nesting, multiple coexisting illnesses, and functional impairments. These factors significantly increase the socioeconomic burden associated with healthy aging (2), placing sustained pressure on pension payments, healthcare service provision, and long-term care systems (3). As potential seniors, middle-aged individuals constitute the primary cohort facing aging and warrant attention as society ages. Middle-aged and older adults have higher disease incidence rates. Owing to declining physical function and cognitive ability, coupled with reduced social interaction and outdoor activities after retirement, they face not only social isolation but also heightened loneliness and mental health challenges (4), and their increasing medical expenditure burden has become a critical social issue that requires urgent resolution. Physical activity plays a vital role in promoting health, preventing disease, and reducing the risk of pre-mature mortality. This is particularly true for middle-aged and older adults with multiple chronic conditions and low physical activity levels, where the positive effects are even more pronounced (5). Therefore, against the dual backdrop of rapid population aging and rising medical expenditures, actively exploring the impact of physical exercise on medical expenditures among middle-aged and older adults and its underlying mechanisms—and thereby developing cost-effective strategies for expenditure control and health promotion—has become an urgent and important policy issue that requires investigation.

Existing research indicates that medical expenditures among middle-aged and older adults are influenced by a combination of factors, including internet use (6), chronic diseases (7), health literacy (8), and age and family structure (9), as well as income and health insurance coverage (10, 11), intergenerational support (12), urbanization (13), and environmental exposure (14). In recent years, physical exercise, as a low-cost health investment, has gradually been incorporated into the framework of medical expenditure research. Research has shown that physical exercise can reduce morbidity and mortality, slow aging (15), improve physical function, alleviate depression and anxiety (4, 16), and promote healthy behaviors among spouses (17), thereby reducing outpatient, inpatient, or out-of-pocket medical expenditures. However, in the digital age, digital technologies have permeated every aspect of human life, transforming the way we communicate, learn, work, and even manage our health (18). On the one hand, the rapid development of digital technology has provided the public with convenient access to fitness information, maintained and enhanced cognitive engagement in physical activity, and optimized exercise methods (19). Increased digital literacy is significantly positively correlated with exercise frequency and duration (20). On the other hand, the internet is becoming a vital source of health information and a potential platform for improving disease management, playing an increasingly critical role in health management (6) by helping individuals access health knowledge and medical information and enhancing their understanding of health management (18). Individuals with higher digital literacy obtain formal or informal social support through digital platforms such as social media, which enhances their ability to access and utilize healthcare services and other health-related resources (21). Thus, while existing research has fully demonstrated the association between digital literacy and physical exercise as well as medical expenditures, few studies have examined the role of digital literacy in the impact of physical exercise on medical expenditures.

Based on the above considerations, this study focuses on the impact of physical exercise on medical expenditures among middle-aged and older adults and its underlying mechanisms. It explores the pathways through which physical exercise influences medical expenditures and examines the moderating role of digital literacy in these relationships. The structure of this study is as follows. Section 2 proposes research hypotheses on the basis of theoretical analysis. Section 3 elaborates on the data and methods. Section 4 analyses the empirical results. Section 5 presents a systematic discussion of the research, offers relevant policy recommendations, and identifies the study's limitations. Section 6 clarifies the main conclusions of this study.

## 2 Development of research hypotheses

### 2.1 Physical exercise and medical expenditures

Physical exercise can influence medical expenditure among middle-aged and older adults. First, from the perspective of health capital accumulation. Grossman's health capital theory posits that health serves as both a source of utility and an investable capital stock. Physical exercise is a quintessential proactive health investment that enhances cardiovascular endurance, improves metabolic capacity, and increases muscle strength and balance. These improvements increase an individual's health capital stock and slow the depreciation rate. Long-term physical activity accumulates health capital reserves, delaying or mitigating health risks (22). Simultaneously, exercise serves as a social activity that enhances communication among middle-aged and older adults, reducing loneliness (4). Regular physical activity effectively decreases hospital stays, admission frequency, and total medical expenditures (23), thereby improving health while reducing overall medical expenditures. Second, from the perspective of reduced healthcare demand. As a chronic disease management strategy, regular physical exercise is more cost effective than pharmacological treatments are (24). By enhancing the cognitive function of middle-aged and older adults, regular exercise empowers them to take a more active role in medical decision-making and doctor–patient communication. This improves the self-management of conditions, thereby reducing unnecessary tests, redundant medical visits, and excessive medical expenditures (16). Regular physical activity aids middle-aged and older adults in weight management, promotes healthy dietary habits, and promotes positive lifestyles (25). This approach reduces chronic disease risks and slows functional decline at their source, thereby decreasing reliance on medical and long-term care services. On the basis of these observations, the following research hypothesis is proposed:

**Hypothesis 1:** physical exercise significantly reduces medical expenditure among middle-aged and older adults.

### 2.2 The mediating role of physical and mental health

Physical exercise can improve the physical health of middle-aged and older adults, primarily by maintaining physical function, preventing disease, and delaying the onset of functional decline. First, as a planned and highly repetitive health-promoting activity, physical exercise enhances muscle strength, improves cardiopulmonary function, and regulates metabolic and neuroendocrine processes, thereby increasing energy and endurance, alleviating fatigue, and improving overall physical function (16, 26). Second, middle-aged and older adults are more susceptible to chronic diseases owing to factors such as declining physiological function and reduced metabolic rates. Regular exercise can effectively reduce the risk of chronic diseases, such as hypertension, cancer, diabetes, and coronary heart disease (27). Furthermore, as people age, they often experience issues such as weakened lower body muscle strength, impaired balance, and reduced joint flexibility (28). Physical exercise can increase bone density in middle-aged and older adults, reduce the risk of osteoporosis (29), and effectively improve functional performance in areas such as balance and flexibility, thereby reducing the risk of falls and fall-related injuries (30).

Physical exercise can improve mental health among middle-aged and older adults. Regular physical exercise significantly alleviates anxiety and depression and enhances subjective wellbeing (15). Specifically, physical exercise regulates the function of the neuroendocrine system; promotes the secretion of neurotransmitters such as brain-derived neurotrophic factor (BDNF), endorphins, and serotonin in the brain (31); enhances neuroplasticity and cognitive function (32); and triggers adaptive mechanisms that reduce the perception of negative emotions, thereby alleviating depression, anxiety, and cognitive decline (33). Furthermore, as a behavior embedded in social contexts, physical exercise has been widely shown to enhance the diversity of an individual's social network, increase the frequency of interactions, and optimize the network structure (27). For middle-aged and older adults, regular physical exercise can promote social interaction through participation in social groups, foster a sense of belonging, reduce feelings of loneliness (34), alleviate stress levels, and reduce negative emotions such as anxiety and depression (35).

Physical exercise can effectively reduce medical expenditures by optimizing an individual's physical and mental health. Its mechanism of action can be explained from three dimensions: physical illness, mental illness, and comorbidity. From the perspective of physical illness, the coexistence of multiple chronic conditions limits an individual's ability to live independently (36), complicates clinical management, and leads to increased utilization of medical resources and costs (37). Good physical health helps reduce the likelihood of chronic and acute illnesses and decreases the incidence of nutrition- and lifestyle-related diseases, such as obesity, osteoporosis, and hyperlipidemia (16). It can trigger anti-inflammatory responses, enhance antioxidant defenses, and improve insulin sensitivity and metabolic efficiency (38), thereby reducing the need for acute interventions, inpatient care, and long-term medications. From the perspective of mental health, exercise serves as an effective cross-diagnostic adjunctive treatment in mental health outpatient care (39). Early exercise intervention for mental health issues can reduce the future risk of psychiatric medication use by 15% (39), thereby alleviating the economic burden on mental health service systems. From the perspective of comorbidities, physical and mental illnesses often have a reciprocal causal relationship, leading to comorbid conditions such as diabetes and depression or coronary heart disease and anxiety (40, 41). Physical exercise breaks the vicious cycle of comorbidity by simultaneously improving metabolic function and emotional regulation, thereby reducing the risk of repeated hospital visits and inpatient treatment for patients with comorbidities and minimizing the multiple uses of medical resources. On this basis, the following research hypothesis is proposed.

**Hypothesis 2:** physical exercise indirectly reduces medical expenditure for middle-aged and older adults by improving their physical health.
**Hypothesis 3:** physical exercise indirectly reduces medical expenditure for middle-aged and older adults by improving their mental health.

### 2.3 Moderating role of digital literacy

Digital literacy enhances the ability of middle-aged and older adults to access and utilize sports resources, health information, and medical services, thereby strengthening the role of physical exercise in reducing medical expenditures. This can be analyzed across three dimensions: digital operational, digital professional, and digital social literacy.

First, digital operational literacy primarily reflects the extent to which middle-aged and older adults apply basic digital skills; the higher the level of application is, the more effectively they can utilize digital technologies to support their work and daily lives (42). Digital skills and knowledge are key factors influencing older adults' participation in mobile and web-based exercise interventions, as they determine the acceptability and sustained participation in these programs (43). Exercise-related risks, such as falls, cardiovascular events, and exacerbations of chronic conditions, are major factors hindering sustained physical activity among middle-aged and older adults. Through smart devices and health apps, this demographic can access precise exercise guidance, monitor physiological indicators, and manage exercise-related risks (44), thereby improving the sustainability of healthy behaviors and the quality of chronic disease management (45), ultimately reducing the overall demand for healthcare services.

Second, digital professional literacy primarily manifests as the ability of middle-aged and older adults to utilize digital resources and technologies in learning and work contexts (18). Health economics theory indicates that human capital, such as education and skills, can increase the efficiency of health investments, thereby influencing health behavior choices and outcomes. In an information technology environment, improvements in learning capacity, cognitive updating ability, and labor adaptability not only help delay functional decline caused by health deterioration but also strengthen health investment awareness and long-term health planning capabilities (46). The use of digital technologies can significantly reduce the risk of dementia and the rate of cognitive decline among middle-aged and older adults (47), increase individuals' awareness of health risks and the benefits of prevention, and encourage them to engage in proactive health management through regular exercise (44, 48), thereby reducing their reliance on high-cost medical interventions.

Finally, digital social literacy primarily reflects the ability of middle-aged and older adults to use digital tools for information exchange, social interaction, and network building, thereby obtaining financial and informational support from social networks (20). Social support and peer motivation are key factors influencing participation in physical activity (49). Digital social platforms can enhance the social norms and peer motivation effects of healthy behaviors, thereby increasing the consistency and retention of physical exercise (50). This leads to greater emotional support, reduced loneliness and depression, and a decrease in the demand for healthcare services related to mental health issues, which in turn further reduces medical expenditures (51). On this basis, the following research hypothesis is proposed.

**Hypothesis 4:** digital literacy enhances the role of physical exercise in curbing medical expenditures among middle-aged and older adults.

**Figure 1:**
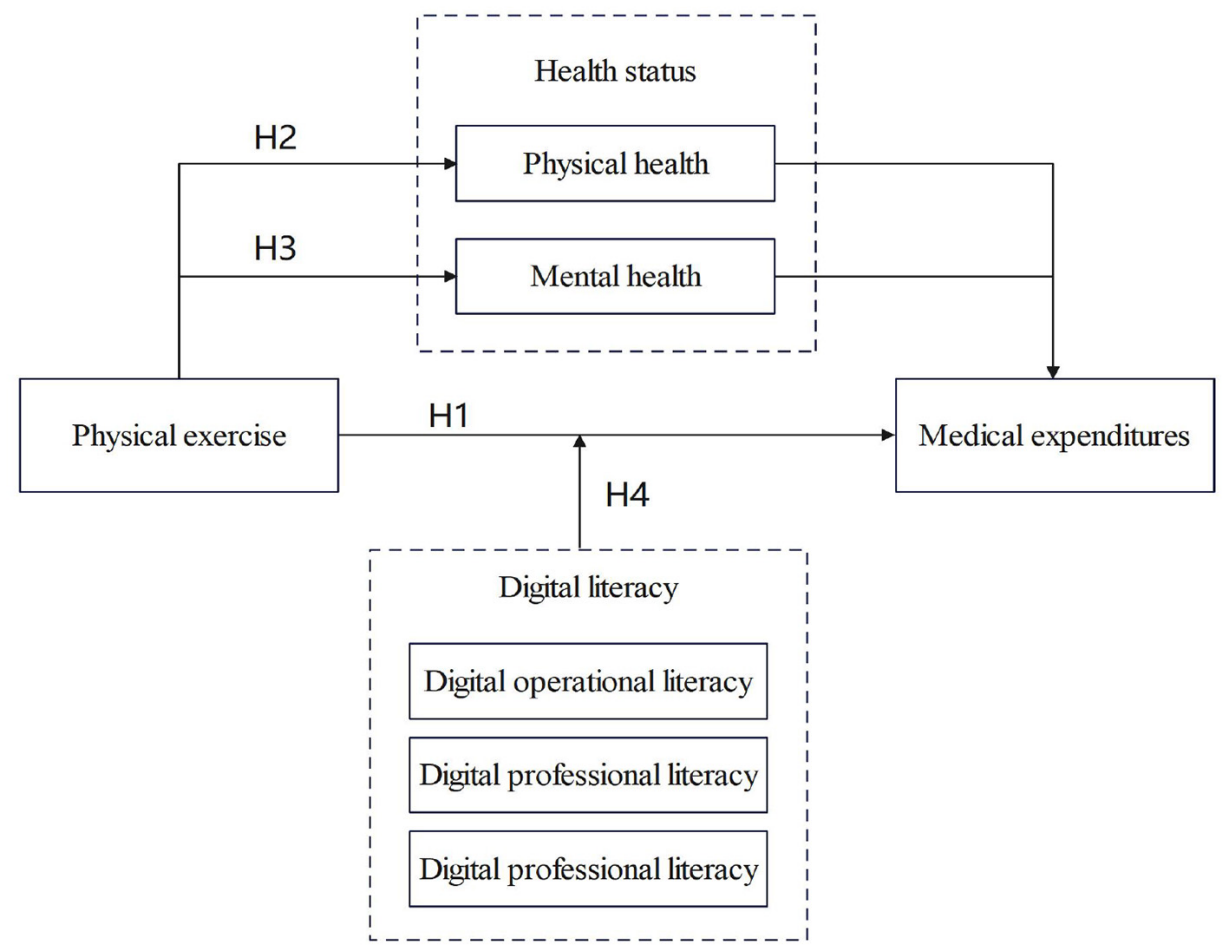
Theoretical framework.

A diagram of the theoretical analysis based on the above analyses is shown in Figure 1.

## 3 Data source and methodology

### 3.1 Data and study sample

The study participants were selected from the China Family Panel Studies (CFPS), a large-scale, nationwide, and continuous social tracking survey project conducted every 2 years by the China Social Science Survey Center of Peking University. The CFPS database covers economic, demographic, educational, and health conditions in 25 provinces in China. The latest data in the CFPS database are from 2022. Owing to differences in the measurement methods for various variables in 2018, this study selected data from 2020–2022 as the research sample. Individual and household questionnaires were matched using personal and household identification codes. The CFPS database consists of family, adult, and child databases. We used the adult database for analysis because this study focused on middle-aged and older adults with chronic diseases. Province-level data were derived from the China Statistical Yearbook and provincial statistical yearbooks. Following Xing et al. (6), we selected those aged over 45 years with chronic diseases. After removing missing data and outlier samples, we constructed a panel database comprising 4,820 samples to examine the relationship between physical exercise and medical expenditures among these populations.

### 3.2 Variable definition

#### 3.2.1 Dependent variable

The dependent variable was medical expenditure among middle-aged and older adults. Previous studies typically employed per capita medical expenditure from the China Statistical Yearbook or the China Health Statistics Yearbook as a measurement indicator. However, these indicators reflect only the average level of individual medical expenditures and fail to capture differences between individuals and groups (52–54). The CFPS questionnaire includes three items measuring medical expenditures: hospitalization expenses (in the past 12 months, including reimbursed and expected reimbursable portions, how much did you spend in total for hospitalization?). Outpatient expenses (in the past 12 months, including reimbursed and expected reimbursable portions, how much did you spend in total for illnesses or injuries?); and out-of-pocket medical expenses (in the past 12 months, how much did you personally pay directly for illnesses or injuries?). These indicators have been empirically validated in existing studies and are considered to have good validity and reliability and are capable of reflecting an individual's level of medical expenditure (6, 17, 55). In this study, “out-of-pocket medical expenses” were used to measure the level of medical expenditure among middle-aged and older adults. These data were log-transformed; for cases where medical expenditures were zero, a value of one was added before taking the logarithm.

#### 3.2.2 Independent variable

The explanatory variable was physical exercise among middle-aged and older adults. Following the methodology of Huang et al. (56), the item “frequency of physical exercise (times)” from the CFPS questionnaire was selected to construct a binary dummy variable for sports participation, “whether engaged in physical exercise”, on the basis of respondents' exercise frequency over the past week. Samples with ≥1 weekly exercise session were coded as 1 (engaged in physical exercise), while those without were coded as 0 (not engaged in physical exercise).

#### 3.2.3 Mediating variables

The mediating variables in this study are divided into two parts: physical health and mental health. The specific measurement methodology followed the approach outlined by Yang et al. (18). First, physical health uses personal self-assessments of physical condition, measured by the following question: how do you think your health is?”, with the following specific values: “no health = 1”, “general health = 2”, “fair health = 3”, “quite health = 4”, and “very health = 5”. Second, mental health encompasses an individual's depression level, social cognition, and emotional experience. We employed the Center for Epidemiologic Studies Depression Scale (CES-D) as the assessment tool (57). This scale has been extensively used in clinical settings for diagnosing depressive symptoms and in the literature for mental health evaluations, demonstrating its scientific rigor and reliability. The selected CES-D items were incorporated into the CFPS questionnaire, including “I feel down”, “I find everything a lot of effort”, “I have trouble sleeping”, “I feel lonely”, “I feel sad”, “I feel like life is hopeless”, “I feel cheerful”, and “I feel happy”. Responses to these eight items are rescored: the first six items are scored in reverse, and the scores from all eight items are summed, yielding a range from 0 to 24. Higher scores indicate better mental health and lower depression levels; conversely, lower scores indicate poorer mental health and higher depression levels.

#### 3.2.4 Moderating variables

The moderating variable is the digital literacy of middle-aged and older adults. Digital literacy is the set of skills and knowledge that helps individuals live, learn, and work in a digital society, such as accessing, communicating, evaluating, and creating information through digital technologies (18). Domestic and international studies have developed distinct digital literacy assessment frameworks from varying perspectives. For instance, on the basis of data from China's Rural Revitalization Survey, Li et al. (26) designed a digital literacy indicator system encompassing four dimensions: digital access literacy, digital access capability literacy, digital application capability literacy, and digital awareness literacy. Drawing on data from the China Family Panel Studies, Zhang et al. (58) constructed a digital literacy indicator system centered on two dimensions: digital capability and digital awareness; Song et al. (59) developed a digital literacy assessment framework encompassing four dimensions: digital operational literacy, digital professional literacy, digital social literacy, and digital information literacy; and Yang et al. (18) constructed an indicator system based on three dimensions: digital general literacy, digital life literacy, and digital professional literacy. While academic consensus on the specific indicator framework for digital literacy remains elusive, there is broad agreement that digital literacy constitutes the foundational capability that enables individuals to successfully integrate and thrive in the rapidly evolving digital era. In summary, drawing upon the approaches of the aforementioned scholars and integrating the practical daily lives of middle-aged and older adults, this study constructed a digital literacy indicator system based on three dimensions: digital operational literacy, digital professional literacy, and digital social literacy (see Table 1). After this indicator evaluation system is established, the entropy method is applied for weighted calculations. The resulting scores measure an individual's digital literacy level, with higher scores indicating greater proficiency.

**Table 1:** The digital literacy index.

Dimension	Indicator	Variable description
Digital operational literacy	Access to internet by mobile device	1 = yes, 0 = no
	Access to internet by computer	1 = yes, 0 = no
Digital professional literacy	Have you used online learning in the past week?	1 = yes, 0 = no
	How important is the internet for work?	1~5, from low to high
	How important is the internet for Study?	1~5, from low to high
Digital social literacy	Have you used WeChat in the past year?	1 = yes, 0 = no
	Have you watched short videos or live streaming programs on online platforms?	1 = yes, 0 = no
	How important is the internet for staying connected with your family and friends?	1–5, from low to high

The three dimensions of digital literacy (technical, professional, and social) comprise multiple sub-indicators with varying data characteristics. This study employs the entropy method to comprehensively measure digital literacy across different dimensions. The specific steps are as follows.

First, the extreme value method was used to normalize each indicator of digital literacy.

For positive indicators, the calculation formula (Equation 1) is as follows:


$$\begin{array}{l} \begin{array}{c} X_{ij}^{*}=\frac{\left(X_{ij}-X_{\min}\right)}{\left(X_{\max}-X_{\min}\right)},\;i\;=\;1,2,\cdots \;,m;\;j\;=\;1,2,\cdots \;,n \end{array} \end{array}$$ (1)

Next, the entropy method is used to determine the weight ω_*j*_ for each indicator. The following formula (Equation 2) is then applied to determine the exponent for each indicator:


$$\begin{array}{lll} P_{i}\left(t_{k}\right)\; & = & \;\overset{n}{\underset{j\;=\;1}{\sum }}\omega_{j}\;\times \;X_{ij}\left(t_{k}\right),k\;=\;1,2,\cdots \;, \end{array}$$

$$\begin{array}{lll} T;i\; & = & \;1,2,\cdots \;,m;j\;=\;1,2,\cdots \;,n \end{array}$$ (2)

Finally, the exponents of all the indicators were summed to calculate the overall digital literacy index. The digital operational literacy, digital professional literacy, and digital social literacy indices were calculated using the same method as the overall digital literacy index.

#### 3.2.5 Control variable

In this study, the following control variables were selected to mitigate endogeneity issues arising from omitted variables: (1) individual characteristics, primarily including age, marital status, employment, education, chronic diseases, smoking status, and drinking status; (2) household characteristics, primarily including household size and household income; and (3) regional characteristics, primarily including per capita GDP and the medical consumer price index. The specific measurements are presented in Table 2.

**Table 2:** Variable definitions.

Variable type	Variable name	Variable definition
Dependent variable	Medical expenditure	Household out-of-pocket medical expenditure, using logarithms
Independent variable	Physical exercise	Participated in physical exercise in the past week, yes = 1, no = 0
Mediating variable	Physical health	Self-assessed health statuses assigned values of 1 to 5 indicating poor to excellent
	Mental health	Calculated based on the 8-question CES-D scale
Moderating variables	Digital literacy	Comprehensive indicators
Control variable	Age	Respondents' actual age in the survey year
	Marriage	Married is assigned a value of 1, and 0 otherwise
	Employ	Employed is assigned a value of 1, and 0 otherwise
	Education	Illiterate or semiliterate = 1; primary school = 2; junior high school = 3; high school = 4; college degree or above = 5
	Chronic diseases	Suffer from chronic diseases, yes = 1, no = 0
	Smoke	Smoking is assigned a value of 1, and 0 otherwise
	Drink	Drinking is assigned a value of 1, and 0 otherwise
	Household size	Number of members in the household
	Household income	Household annual income, using logarithms
	PGDP	Per capita regional gross domestic product (RMB per person)
	MCPI	Consumer price index for healthcare (base year = 100)

#### 3.2.6 Descriptive statistics

Table 3 reports the descriptive statistics for the key variables. First, the mean medical expenditure is 1,792.87, with a standard deviation of 4,520.703, ranging from 0 to 30,500. These findings indicate that medical expenditure levels among middle-aged and older adults are generally low and significantly vary. Second, the average self-rated physical health score is 2.938, which falls within the moderate range of 1–5 points. This finding indicates that most individuals assessed their health status as moderate and considered themselves “relatively healthy”. Third, the average mental health score is 18.632. The average score for depressive tendencies is 5.368, which corresponds to 22.37% of the maximum possible score. Given that the mental health scale uses reverse coding, a lower score indicates poorer mental health. Following the methodology of Yang et al. (18), we use the 20th percentile score as the cutoff score of depression. By calculating the proportion of respondents with scores below the 20th percentile, we determine that the prevalence of depressive symptoms is 15.87%. This indicates that Chinese respondents generally enjoy good mental health and exhibit low levels of depressive tendencies. Fourth, the average digital literacy level is 0.231, which was less than 0.5 and ranged from 0.019 to 1, with a standard deviation of 0.238. These findings indicate that the digital literacy level among middle-aged and older adults is generally low and varies significantly.

**Table 3:** Descriptive statistics of the main variables.

Variable type	Variable name	Mean	Std. dev	Min	Max
Dependent variable	Medical expenditure	1,792.87	4,520.703	0	30,500
Independent variable	Physical exercise	0.354	0.478	0	1
Mediating variables	Physical health	2.938	1.137	1	5
	Mental health	18.632	4.087	1	24
Moderating variable	Digital literacy	0.231	0.238	0.019	1

### 3.3 Methodology

#### 3.3.1 Empirical strategy

Since the dependent variable of medical expenditures among middle-aged and older adults is continuous and passes the Hausman test, a fixed-effects model was employed to examine the impact of physical exercise on medical expenditures. The specific model (Equation 3) specifications are as follows:


$$\begin{array}{l} \begin{array}{c} HE_{it}\;=\;\alpha_{0}+\alpha_{1}{Sport}_{it}+\alpha_{n}C_{it}+y_{t}+p_{i}+\varepsilon_{it} \end{array} \end{array}$$ (3)

Among these, *HE*_*it*_ represents the dependent variable of medical expenditures for older adults, denoting the medical expenditure of individual *i* at time *t*; *Sport*_*it*_ denotes the independent variable of participation in physical exercise, indicating whether individual *i* participated in physical exercise at time *t*; *C*_*it*_ represents a series of control variables (including age, marital status, employment status, education level, chronic diseases, household income, etc.); α_0_ denotes the constant term; *y*_*t*_ and *p*_*i*_ denote the time effect and individual fixed effect, respectively; ε_*it*_ is the random disturbance term; α_*n*_ represents the set of regression coefficients for the control variables; and α_1_ is the key parameter measuring the impact of *Sport*_*it*_ on *HE*_*it*_. If the coefficient α_1_ is significantly negative, it indicates that participation in physical exercise suppresses medical expenditures among middle-aged and older adults.

#### 3.3.2 Mediation effect model

To further explore the underlying mechanisms through which physical exercise influences medical expenditures among middle-aged and older adults, this study employs a three-step approach (60) that combines Sobel tests and Bootstrap methods to examine the mediating effects. The specific steps are as follows: first, model (3) was used to examine the impact of physical exercise on medical expenditures among middle-aged and older adults. Second, model (4) was employed to assess the effect of physical exercise on the mediating variable. Finally, model (5) was used to evaluate the influence of physical exercise on medical expenditures after controlling for the mediating variable. The specific mediation effect model is set as follows:


$$\begin{array}{l} \begin{array}{c} M_{it}\;=\;\beta_{0}+\beta_{1}Sport_{it}+\beta_{n}C_{it}+y_{t}+p_{j}+\varepsilon_{it}\; \end{array} \end{array}$$ (4)

$$\begin{array}{l} \begin{array}{c} {HE}_{it}\;=\;\gamma_{0}+\gamma_{1}Sport_{it}+\gamma_{2}M_{it}+\gamma_{n}C_{it}+y_{t}+p_{j}+\varepsilon_{it}\; \end{array} \end{array}$$ (5)

Among these, *M*_*it*_ represents the set of mediating variables, namely, physical and mental health, while the remaining variables are identical to those in model (3).

#### 3.3.3 Moderation effect model

Furthermore, to examine whether digital literacy among middle-aged and older adults enhances the inhibitory effect of physical exercise on medical expenditures, the following moderation effect model (Equation 6) was established:


$$\begin{array}{lll} HE_{it}\; & = & \;\delta_{0}+\delta_{1}Sport_{it}+\delta_{2}dl_{it}+\delta_{3}\left(Sport_{it}\;\times \;dl_{it}\right)+\delta_{n}C_{it} \end{array}$$

$$\begin{array}{ll} + & y_{t}+p_{j}+\varepsilon_{it} \end{array}$$ (6)

Here, *dl*_*it*_ denotes the moderator variable (digital literacy), and the remaining variables are identical to those in model (3).

## 4 Results

### 4.1 Baseline regression results

Table 4 presents the baseline regression results. Columns (1) to (4) show the effects of physical exercise on medical expenditures under different scenarios. Column (1) does not include control variables, whereas columns (2) through (4) include control variables at the individual, household, and regional levels, respectively. The results in columns (1) to (4) indicate that physical exercise significantly reduces medical expenditures among middle-aged and older adults, regardless of whether control variables are included. Specifically, when both control variables and fixed effects are controlled for [as shown in column (4)], the regression coefficient for physical exercise is −0.983 (*p* < 0.01), indicating that hypothesis H1 is valid. In practical terms, the regression results in column (4) indicate that, controlling for other factors, middle-aged and older adults who engage in physical exercise have medical expenditures that are 0.983 units lower than those who do not engage in physical exercise. These findings confirm that physical exercise can significantly reduce medical expenditure among middle-aged and older adults.

**Table 4:** Baseline regression results.

Variables	Medical expenditure			
	(1)	(2)	(3)	(4)
Physical exercise	−0.690^**^	−0.959^***^	−0.980^***^	−0.983^***^
	(−2.410)	(−3.432)	(−3.499)	(−3.500)
Age		−0.492	−0.459	−0.442
		(−0.380)	(−0.355)	(−0.340)
Marriage		−0.956	−0.910	−0.904
		(−1.252)	(−1.171)	(−1.162)
Employ		1.087^***^	−1.119^***^	−1.112^***^
		(−2.749)	(−2.822)	(−2.799)
Education		1.047^**^	1.014^**^	1.009^**^
		(2.308)	(2.229)	(2.213)
Chronic diseases		2.369^***^	2.377^***^	2.373^***^
		(8.255)	(8.275)	(8.244)
Smoke		−0.816	−0.824	−0.815
		(−1.549)	(−1.562)	(−1.543)
Drink		−0.253	−0.245	−0.253
		(−0.693)	(−0.668)	(−0.689)
Household size			−0.116	−0.112
			(−0.752)	(−0.727)
Household income			0.217	0.216
			(1.089)	(1.082)
PGDP				0.508
				(0.382)
MCPI				0.719
				(0.123)
Constant	3.917^***^	29.023	25.346	15.467
	(30.896)	(0.416)	(0.363)	(0.195)
Year fixed	Yes	Yes	Yes	Yes
Individual fixed	Yes	Yes	Yes	Yes
N	4,820	4,820	4,820	4,820
R^2^	0.017	0.103	0.104	0.104
F	7.834	11.477	9.507	8.039

^***^p < 0.01, ^**^p < 0.05, ^*^p < 0.10.

### 4.2 Robustness tests

#### 4.2.1 Propensity score matching

To accurately identify the impact of “physical exercise”, the study requires the construction of experimentally similar treatment and control groups within a counterfactual framework. To this end, propensity score matching (PSM) was employed to mitigate endogeneity issues, such as sample selection bias, thereby enhancing the reliability of the findings. Building on prior research, we employed 1:2 nearest-neighbor matching and kernel matching, using individual, household, and regional-level control variables as covariates to ensure that no systematic differences existed between the experimental and control groups. After excluding a small number of unmatched samples, we retested them using model (3), with the regression results shown in columns (1) and (2) of Table 5. The regression coefficient for physical exercise remained significantly negative, confirming the robustness of the findings.

**Table 5:** Robustness test.

Variables	Medical expenditure					
	(1)	(2)	(3)	(4)	(5)	(6)
Physical exercise	−1.679^***^	−0.995^***^		−0.977^***^	−0.944^***^	−0.144^***^
	(−3.988)	(−3.538)		(−3.459)	(−3.274)	(−2.727)
L. Physical exercise			−1.269^***^			
			(−4.990)			
Constant	74.723	22.304	26.664	5.367	8.169	10.507
	(1.290)	(0.280)	(0.224)	(0.063)	(0.100)	(0.132)
Control	Yes	Yes	Yes	Yes	Yes	Yes
Year fixed	Yes	Yes	Yes	Yes	Yes	Yes
Individual fixed	Yes	Yes	Yes	Yes	Yes	Yes
Province fixed	No	No	No	Yes	No	No
N	2,552	4,787	909	4,820	4,820	4,820
R^2^	0.168	0.108	0.271	0.116	0.098	0.100
F	4.861	8.266	27.790	4.623	7.519	7.631

^***^p < 0.01, ^**^p < 0.05, ^*^p < 0.10.

#### 4.2.2 Lagged independent variable

One source of endogeneity stems from bidirectional causality. Physical exercise among middle-aged and older adults can influence their medical expenditures; however, simultaneously, increased medical expenditure among these individuals may also encourage them to actively participate in fitness-enhancing physical activities. To overcome the potential estimation bias from this bidirectional causality, this study introduces a lagged variable of physical exercise as an independent variable, regressing exercise one period ahead. As shown in column (3) of Table 5, the regression coefficient for exercise is −1.269 (*p* < 0.01), confirming the robustness of the above findings. In practical terms, when controlling for other factors, middle-aged and older adults who engage in physical exercise have medical expenditures that are 1.269 units lower than those who do not.

#### 4.2.3 Consider omitted variables

Another source of endogeneity is the omitted variables. Beyond controlling for variables, the regression model in Table 4 accounts for individual-level characteristics that are difficult to measure (such as marital status, education level, and chronic disease status) affecting medical expenditures among middle-aged and older adults by incorporating individual fixed effects. It also controls for time series effects. However, regional differences in medical insurance policies and reimbursement systems also influence medical expenditures in this demographic. To address this issue, this study retains individual and time fixed effects in the baseline regression model while further incorporating provincial fixed effects. This adds an additional layer of control for regional factors affecting medical expenditures among middle-aged and older individuals. As shown in column (4) of Table 5, the regression coefficient for physical exercise is −0.977 (*p* < 0.01), confirming the robustness of the above findings.

#### 4.2.4 Change variables

The benchmark regression estimates may be subject to bias because of measurement differences in variables such as the dependent variable (medical expenditure) and the independent variable (physical exercise). First, following the approach of Fan and Yin (61), total medical expenses for middle-aged and older adults are selected as a proxy variable for medical expenditure and log-transformed. The results of using model (3) for regression testing are shown in column (5) of Table 5. The estimated coefficient for physical exercise is −0.944 (*p* < 0.01). Second, we altered the measurement of the independent variable “physical exercise” by replacing the binary dummy variable “whether participants in physical exercise” with the frequency of exercise. On the basis of the results of the CFPS questionnaire, the specific values are as follows: “Never participates = 0”, “Less than once per month on average = 1”, “More than once per month on average but less than once per week = 2”, “1–2 times per week on average = 3”, “3–4 times per week on average = 4”, “5 times or more per week on average = 5”, “Once per day = 6”, and “Twice or more per day = 7”. Rerunning the regression analysis using model (3) yields the results shown in column (6) of Table 5. The estimated coefficient for physical exercise is −0.144 (*p* < 0.01), confirming the robustness of the findings above.

### 4.3 Mechanism analysis

This study uses model (Equation 4) and model (Equation 5) to test the mediating effect of physical and mental health. First, the test results in columns (1) and (2) of Table 6 demonstrate the mediating effect of physical health on the relationship between physical exercise and medical expenditures among middle-aged and older adults. Column (1) shows that the estimated coefficient for the impact of physical exercise on physical health is 0.151 (*p* < 0.05). In practical terms, controlling for other factors, middle-aged and older adults who engage in physical exercise have a physical health score that is 0.151 units higher than that of those who do not exercise. The analysis of the estimates in column (2) reveals that the estimated coefficient for physical health is −0.520 (*p* < 0.01). The estimated coefficient for physical exercise was −0.904 (*p* < 0.01), which was lower than the baseline regression coefficient (−0.983). These findings indicate that physical exercise reduces medical expenditures by enhancing physical health among middle-aged and older adults. Thus, hypothesis H2 is valid.

**Table 6:** Mechanism analysis results.

Variables	(1)	(2)	(3)	(4)
	Physical health	Medical expenditure	Mental health	Medical expenditure
Physical exercise	0.151^**^	−0.904^***^	0.591^**^	−0.932^***^
	(2.018)	(−3.243)	(2.148)	(−3.322)
Physical health		−0.520^***^		
		(−4.176)		
Mental health				−0.086^**^
				(−2.519)
Sobel z-stat/Bootstrap CI	−11.270^***^		−7.227^***^	
	[−0.458, −0.331]		[−0.211, −0.121]	
Constant	27.316	29.679	50.598	19.797
	(1.293)	(0.376)	(0.649)	(0.250)
Control	Yes	Yes	Yes	Yes
Year fixed	Yes	Yes	Yes	Yes
Individual fixed	Yes	Yes	Yes	Yes
N	4,820	4,820	4,820	4,820
R^2^	0.023	0.122	0.027	0.111
F	1.618	8.847	1.890	7.962

^***^p < 0.01, ^**^p < 0.05, ^*^p < 0.10.

Second, the test results in columns (3) and (4) of Table 4 demonstrate the mediating effect of mental health on physical exercise and medical expenditures among middle-aged and older adults. Column (3) shows that the estimated coefficient for the effect of physical exercise on mental health is 0.591 (*p* < 0.05). In practical terms, when other factors are controlled for, middle-aged and older adults who engage in physical exercise have a mental health score that is 0.591 units higher than that of those who do not exercise. Analysis of the estimates in column (4) reveals that the estimated coefficient for mental health is −0.086 (*p* < 0.01). The estimated coefficient for physical exercise was −0.932 (*p* < 0.01), which was lower than the baseline regression coefficient (−0.983). These findings indicate that physical exercise reduces medical expenditure among middle-aged and older adults by promoting mental health. Thus, hypothesis H3 is valid.

### 4.4 Moderation effect analysis

This study verifies the presence of a moderating effect by including interaction terms between the moderator and independent variables in model (Equation 6). The regression results are presented in column (1) of Table 7. The interaction coefficient between physical exercise and digital literacy is −1.876 (*p* < 0.05). This finding indicates that when other conditions are held constant, higher levels of digital literacy among middle-aged and older adults strengthen the inhibitory effect of physical exercise on medical expenditures. Thus, hypothesis H4 is valid.

**Table 7:** Moderation effect results.

Variables	Medical expenditure			
	(1)	(2)	(3)	(4)
Physical exercise	−0.942^***^	−0.959^***^	−0.916^***^	−0.979^***^
	(−3.368)	(−3.423)	(−3.267)	(−3.480)
Digital literacy	−1.211^**^			
	(−2.031)			
Physical exercise × digital literacy	−1.876^**^			
	(−2.034)			
Digital operational literacy		−0.991^**^		
		(−2.474)		
Physical exercise × digital operational literacy		0.164		
		(0.256)		
Digital professional literacy			−0.233	
			(−0.597)	
Physical exercise × digital professional literacy			−1.834^***^	
			(−2.851)	
Digital social literacy				0.058
				(0.159)
Physical exercise × digital social literacy				0.050
				(0.072)
Constant	27.116	24.489	18.637	16.205
	(0.342)	(0.308)	(0.235)	(0.204)
Control	Yes	Yes	Yes	Yes
Year fixed	Yes	Yes	Yes	Yes
Individual fixed	Yes	Yes	Yes	Yes
N	4,820	4,820	4,820	4,820
R^2^	0.114	0.110	0.113	0.104
F	7.689	7.391	7.626	6.923

^***^p < 0.01, ^**^p < 0.05, ^*^p < 0.10.

Furthermore, to explore the relative contributions of the three dimensions of digital literacy as moderators, this study calculated composite indices for each dimension (the digital operational literacy index, digital professional literacy index, and digital social literacy index) and incorporated them into model (3) for regression analysis. The regression results are presented in columns (2) to (4) of Table 7. The interaction coefficient between digital professional literacy and physical exercise is −1.834 (*p* < 0.01), and the interaction coefficients between digital operational literacy and digital social literacy with physical exercise are insignificant. These results indicate that the moderating effect of physical exercise on medical expenditures can be maximally amplified only when digital literacy manifests in occupation-related application ability.

### 4.5 Heterogeneity analysis

Given the individual differences in gender, lifestyle, and environment, this study conducts subgroup regression analyses across three dimensions—gender, urban–rural location, and educational attainment—to further identify the heterogeneous characteristics of the effect of physical exercise on the suppression of medical expenditures among middle-aged and older adults and to clarify the group boundaries where digital literacy exerts a moderating influence.

#### 4.5.1 Heterogeneity of sex

We divided the sample into male and female groups on the basis of sex and separately applied them to model (3) to further explore the differences in the relationship between digital literacy and the association between physical exercise and medical expenditures among middle-aged and older adults across sex. The results are presented in columns (1) and (2) of Table 8. Among men, the interaction coefficient between physical exercise and digital literacy was −2.788 (*p* < 0.05). This finding indicates that digital literacy enhances the inhibitory effect of physical exercise on medical expenditures; that is, higher digital literacy strengthens the effect of physical exercise on reducing medical expenditures. But the interaction coefficient was not significant among women, suggesting that digital literacy did not have a notable moderating effect. The above results indicate that the moderating effect of digital literacy has a greater impact on men than on women.

**Table 8:** Heterogeneity test results.

Variables	(1)	(2)	(3)	(4)	(5)	(6)
	Gender		Urban–rural		Education	
	Male	Female	Urban	Rural	High	Low
Physical exercise × digital literacy	−2.788^**^	−1.069	−2.184^*^	0.385	−2.172^*^	−1.148
	(−2.405)	(−0.702)	(−1.904)	(0.220)	(−1.818)	(−0.688)
Physical exercise	−0.844^**^	−1.095^***^	−1.066^***^	−0.450	−1.200^***^	−0.771^*^
	(−2.247)	(−2.597)	(−3.095)	(−0.880)	(−2.724)	(−1.952)
Digital literacy	−1.108	−1.092	−1.846^**^	1.041	−1.335	−1.032
	(−1.464)	(−1.135)	(−2.512)	(0.904)	(−1.600)	(−1.101)
Constant	18.886	−28.471	31.976	−11.288	9.190	−17.604
	(0.435)	(−0.300)	(0.685)	(−0.116)	(0.172)	(−0.402)
Control	Yes	Yes	Yes	Yes	Yes	Yes
Year fixed	Yes	Yes	Yes	Yes	Yes	Yes
Individual fixed	Yes	Yes	Yes	Yes	Yes	Yes
N	2,790	2,030	2,783	2,037	1,580	3,240
R^2^	0.140	0.149	0.142	0.112	0.192	0.084
F	5.885	4.355	6.226	2.767	5.409	3.540

^***^p < 0.01, ^**^p < 0.05, ^*^p < 0.10.

#### 4.5.2 Heterogeneity between urban and rural areas

We divided the sample into urban and rural groups on the basis of household registration status and separately incorporated them into model (3) to further examine the differences in the impact of physical exercise on medical expenditures among middle-aged and older adults across urban and rural contexts, as well as variations in the moderating effect of digital literacy. The results are presented in columns (3) and (4) of Table 8. In the urban group, the interaction coefficient between physical exercise and digital literacy was −2.184 (*p* < 0.1). These findings indicate that digital literacy enhances the inhibitory effect of physical exercise on medical expenditures; that is, higher digital literacy strengthens the effect of physical exercise on reducing medical expenditures. In contrast, the interaction coefficient was not significant for the rural group. These findings suggest that the moderating effect of digital literacy has a greater effect on urban residents than on rural residents.

#### 4.5.3 Heterogeneity of education

We divided the sample into two groups on the basis of educational attainment: below high school and high school or above. Each group was separately incorporated into model (3) to further examine differences in the impact of physical exercise on medical expenditures among middle-aged and older adults across varying educational levels, as well as differences in the moderating effect of digital literacy. The results are presented in columns (5) and (6) of Table 8. In the higher education group, the interaction coefficient between physical exercise and digital literacy was −2.172 (*p* < 0.1). These findings indicate that digital literacy enhances the inhibitory effect of physical exercise on medical expenditures; that is, higher digital literacy strengthens the effect of physical exercise on reducing medical expenditures. In contrast, the interaction coefficient was not significant in the lower-education group. These findings indicate that the moderating effect of digital literacy has a greater effect on higher-education group than lower-education group.

## 5 Discussion and recommendations

### 5.1 Discussion

First, this study revealed that physical exercise can significantly reduce medical expenditures for middle-aged and older adults, a finding that is consistent with the conclusions reached by Liu et al. (62). Middle-aged and older adults typically exhibit characteristics such as multiple coexisting conditions and functional impairments, resulting in a greater medical expenditure. Consequently, the health benefits of physical exercise are directly linked to its ability to reduce medical expenditures in this population. Physical exercise can be viewed as a continuous investment in health, reducing the demand for and costs of healthcare services by increasing health capital stock and slowing its depreciation (63, 64). Furthermore, regular physical activity can improve physical function, thereby lowering the incidence of chronic diseases (65).

Second, this study revealed that physical exercise significantly curbs medical expenditures among middle-aged and older adults through improvements in physical and mental health. This is consistent with the findings of Zhu (16). The cost-reducing effect of physical exercise on medical expenditures stems not only from reductions in morbidity and spending but also from its comprehensive enhancement of physical function and psychological resources, which, in turn, influences disease onset and healthcare-seeking behavior. Physical exercise is consistently associated with multidimensional health outcomes among middle-aged and older adults, with particularly significant effects on alleviating depression and anxiety and improving cognitive function (29). The underlying mechanisms involve improvements in cardiopulmonary function, enhanced neuroplasticity, and emotional regulation (66), as well as strengthened social connections and daily functioning (16). These effects, in turn, reduce the risk of functional impairment and complications, lower the risk of acute exacerbations of chronic diseases, and decrease medical expenditures (62).

Third, digital literacy plays a positive role in reducing medical expenditures among middle-aged and older adults through physical exercise. First, middle-aged and older adults with digital literacy are better able to use online platforms effectively to access health information and exercise-related resources (67), thereby improving access to exercise plans and health tracking tools and promoting physical activity participation among this demographic (59). Additionally, digital platforms provide social support mechanisms that help strengthen people's motivation and perseverance in maintaining a regular exercise routine (68). Second, when older adults use digital resources to manage chronic conditions, such as diabetes or hypertension, they can reduce complications and hospitalizations, thereby directly lowering their medical expenditures (6).

Fourth, among the three dimensions of digital literacy, only digital professional literacy significantly enhances the effect of physical exercise on curbing medical expenditures. This is consistent with the views of Li et al. (26). Within the digital divide, self-efficacy and cognitive gaps are more closely associated with health behaviors (69); the key lies in shaping health-related cognition and behavioral motivation (70) rather than merely in gaps in access and usage. Digital professional literacy primarily reflects the ability of middle-aged and older adults to utilize digital resources and technologies in learning and work contexts (18), and it can enhance their awareness of health investment and their ability to plan for long-term health (46). Individuals with a high level of digital literacy can use digital platforms to access fitness knowledge and information on chronic disease management, thereby enhancing the scientific rigor and consistency of their exercise routines. This enables them to more effectively convert their exercise into accumulated health capital, which in turn reduces medical expenditures. In contrast, the impact of digital operational literacy on health cognition and behavioral motivation is relatively limited; improvements in key chronic disease indicators and health outcomes often require professional guidance and tailored behavioral interventions (71). Additionally, digital social literacy can influence exercise adherence through emotional support and peer motivation; however, it is prone to issues such as insufficient ability to identify misinformation and false claims, which can trigger anxiety or lead to poor health decisions (72), thereby reducing its buffering effect on medical expenditures.

Fifth, the results of this study revealed that the moderating effect of digital literacy varies significantly by gender, urban–rural background, and educational attainment. These findings suggest that middle-aged and older adults exhibit different patterns of technological adaptation and health benefits. The specific analyses are as follows:

Gender Heterogeneity: the moderating effect of digital literacy was significantly stronger among males than among females. This is consistent with the findings of Song et al. (59). The reasons for this lie primarily in two aspects: opportunity and awareness. In the early stages of internet technology adoption, compared with women, men possessed more social resources and higher educational attainment, which gave them greater opportunities to use the internet (73). Additionally, middle-aged and older men may hold more positive perceptions and attitudes toward physical exercise and place greater emphasis on health management (59), making them more inclined to seek health-related information online and thereby improving their health behaviors.

Urban–Rural Heterogeneity: digital literacy exhibited significantly stronger moderating effects among urban populations than among rural ones. This is consistent with the findings of Li et al. (74). Urban areas have more developed digital infrastructure and higher internet penetration rates, and residents in these areas have relatively higher levels of digital literacy, enabling them to search for and select health services and products (58). This, in turn, improves the efficiency and consistency of exercise and reduces unnecessary or inefficient healthcare spending more effectively. In contrast, the digital divide is more pronounced in rural areas, where residents' digital literacy is relatively low, directly limiting their opportunities to access health information and engage in physical exercise through digital platforms (2).

Educational Heterogeneity: the moderating effect of digital literacy is significantly stronger among higher-educated groups than among lower-educated groups. This is consistent with the findings of Wu et al. (75). Specifically, higher educational attainment typically implies stronger learning abilities and accumulated skills, enabling individuals to more easily understand, apply, and adapt to digital technologies (76) and to utilize digital tools more effectively to access, evaluate, and absorb health knowledge and service resources (74). In contrast, older adults with lower educational attainment face limitations in information filtering, health risk identification, and the use of digital tools, making it difficult for them to translate online exercise guidance, health monitoring, and medical information into sustained physical activity and timely preventive interventions (59).

### 5.2 Policy recommendations

This study examined how physical activity among Chinese residents affects their physical and mental health as well as their subsequent medical expenditures, offering important insights for encouraging nationwide participation in physical fitness, formulating targeted health promotion policies, and improving public health standards.

(1) Promotion of physical activity among middle-aged and older adults should be integrated into a collaborative governance framework that addresses healthy aging and medical expenditures. Preventive exercise interventions should be used to alleviate the burden on the healthcare system, and community fitness facilities, walking and cycling environments, age-friendly exercise spaces, and public exercise guidance services should be improved to lower the institutional and environmental barriers to physical activity participation among this demographic. For example, to address Japan's deepening aging population and rising pressure on long-term care, physical exercise programs for middle-aged and older adults could be integrated into comprehensive community care and long-term care prevention systems.
(2) Efforts should be made to shift the “integration of sports and healthcare” from policy advocacy to practical grassroots-level institutional arrangements. Exercise interventions should be incorporated into primary healthcare services, chronic disease management, and health promotion systems, with general practitioners, rehabilitation therapists, public health professionals, and community sports instructors collaborating to provide services such as exercise assessment, exercise prescriptions, chronic disease follow-up, and psychological support. For example, to address the lack of institutionalized integration of exercise interventions in the UK's primary care system, exercise assessments, exercise referrals, and community follow-ups could be incorporated into routine general practice workflows.
(3) The government should incorporate digital literacy education into public health strategies. It should promote the coordinated implementation of digital capacity building and health promotion, disseminate digital knowledge and skills through multiple channels, and strengthen the ability to access health information and participate in physical activities. Simultaneously, the government should regulate digital health platforms to ensure the accuracy of health information and create a positive digital environment for residents. For example, to address the challenges older adults in Singapore face in adapting to digital health services, their digital literacy can be enhanced through community-based digital training, age-friendly platform modifications, and subsidies for device costs.
(4) Implement tiered and targeted health-promoting policies. For women, residents of rural or underdeveloped areas, individuals with low educational attainment, and groups with limited digital resources, priority should be given to providing low-cost, easy-to-use, and sustainable opportunities for physical activity participation and digital support. Policy accessibility should be enhanced through training, family support, and the design of age-friendly digital products. For example, to address the lack of opportunities for physical activity among residents in rural and remote areas of Australia, as well as the multiple barriers that continue to hinder women's participation, tiered interventions can be implemented through community-based integrated service platforms, mobile outreach programs, and targeted funding for women.

### 5.3 Limitations and future research

This study has several limitations. First, the sample consisted solely of individuals aged 45 years and older, which limited the generalizability of the findings. Future research could enhance the external validity of this study by expanding the sample to include a broader age range. Second, this study relies primarily on data from the China Family Panel Studies (CFPS) and is based on self-reports by respondents or their family members. This approach may be subject to social desirability bias, recall bias, and subjective measurement errors, which could affect the reliability of the findings of this study. Third, the survey data provided by the CFPS pertain specifically to the Chinese population; the impact of physical exercise on medical expenditure among residents of other countries requires further exploration. Fourth, the measurement of physical activity in this study may have been relatively limited and focused primarily on basic indicators such as the frequency of participation without fully considering multidimensional factors such as type, intensity, and duration. Future research should develop a more comprehensive system of physical activity indicators to assess the impact of physical activity on medical expenditures more accurately.

## 6 Conclusion

This study empirically examines the effects and mechanisms of active physical exercise on medical expenditure among middle-aged and older adults. The findings revealed the following: (1) physical exercise can reduce medical expenditures for middle-aged and older adults, with physical and mental health serving as mediating factors; (2) higher levels of digital literacy can enhance the effect of physical exercise on reducing medical expenditures for middle-aged and older adults, with digital professional literacy playing a primary moderating role; and (3) compared with women, rural residents, and those with lower levels of education, the moderating effect of digital literacy is stronger among men, urban residents, and those with higher levels of education.

This study provides an empirical foundation for understanding the impact of physical exercise on medical expenditures among middle-aged and older adults and helps to systematically identify and scientifically evaluate the potential economic benefits of physical exercise within the framework of health economics. In the context of an aging population, health management and medical expenditures for middle-aged and older adults will receive increased attention, providing a scientific basis for formulating relevant policies and interventions. Furthermore, incorporating digital literacy into the mechanism of influence expands the interdisciplinary research perspective at the intersection of health economics and digital governance, thereby enriching the theoretical framework in these fields.

## Data Availability

The datasets presented in this article are not readily available because the original data presented in the study are openly available in China Family Panel Studies (CFPS). Requests to access the datasets should be directed to https://cfpsdata.pku.edu.cn and https://www.isss.pku.edu.cn/cfps/en/data/public/index.htm.
